# Recurrent Acute Coronary Syndromes in a Patient with Idiopathic Thrombocytopenic Purpura

**DOI:** 10.1155/2020/6738348

**Published:** 2020-03-13

**Authors:** Nikolaos Iakovis, Andrew Xanthopoulos, Aikaterini Chamaidi, Michail Papamichalis, Apostolos Dimos, Filippos Triposkiadis, John Skoularigis

**Affiliations:** Department of Cardiology, University General Hospital of Larissa, Larissa, Greece

## Abstract

A 53-year-old man was admitted to a peripheral hospital with the diagnosis of acute myocardial infarction without ST elevation. Due to the concomitant presence of first-diagnosed thrombocytopenia (platelet count 50.000/*μ*L), it was decided to be treated conservatively with clopidogrel. Five days later, he developed an acute myocardial infarction with ST elevation (STEMI) and was transferred to our department for primary percutaneous coronary intervention (PCI). Coronary angiography revealed three-vessel disease. The left anterior descending lesion was considered culprit, and PCI was successfully performed using a drug-eluting balloon. This approach was considered safer due to the risk of intolerance of prolonged dual antiplatelet therapy in case of stent implantation. Indeed, four days later, aspirin was discontinued, and the patient remained only on clopidogrel due to a platelet fall. Meanwhile, idiopathic thrombocytopenic purpura (ITP) was diagnosed by hematology consultation, and specific ITP treatment was initiated. Seven days following the procedure, the patient was transferred to the Hematology clinic, where a continuous rise of platelet count up to 115.000/*μ*L while on clopidogrel was observed, and he was discharged from the hospital asymptomatic. Unfortunately, twenty days later, the patient died of a lung infection. In ITP patients with STEMI, primary PCI with drug-eluting balloon angioplasty may be a reasonable approach.

## 1. Introduction

Idiopathic thrombocytopenic purpura (ITP) is an autoimmune disorder characterized by a low platelet count predisposing to bleeding but paradoxically associated with increased risk of acute coronary syndromes (ACS) [1–3]. Herein, we report the case of a 53-year-old man with first-diagnosed ITP and recurrent ACS, treated with stentless primary percutaneous coronary intervention and antiplatelet drug administration.

## 2. Case Presentation

**Figure 1:**
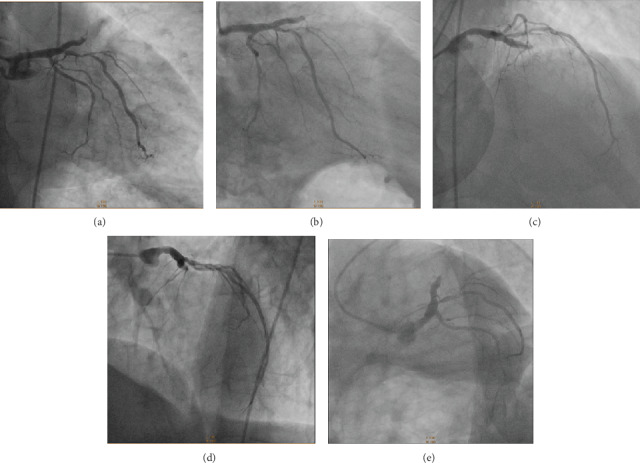
A total occlusion of the left anterior descending coronary artery (LAD), high-grade proximal stenosis in the first diagonal branch (90%), and a diffuse atherosclerotic left circumflex coronary artery (LCx) are depicted in RAO caudal (a, b), RAO cranial (c), LAO cranial (d), and LAO caudal (e) projections. RAO = right anterior oblique; LAO = left anterior oblique.

**Figure 2:**
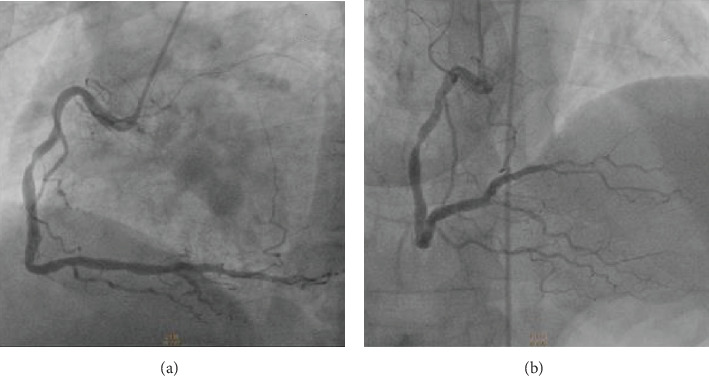
A moderate-severe stenosis (70%) in the middle dominant right coronary artery (RCA) is shown in LAO caudal (a) and RAO cranial (b) projections. RAO = right anterior oblique; LAO = left anterior oblique.

**Figure 3:**
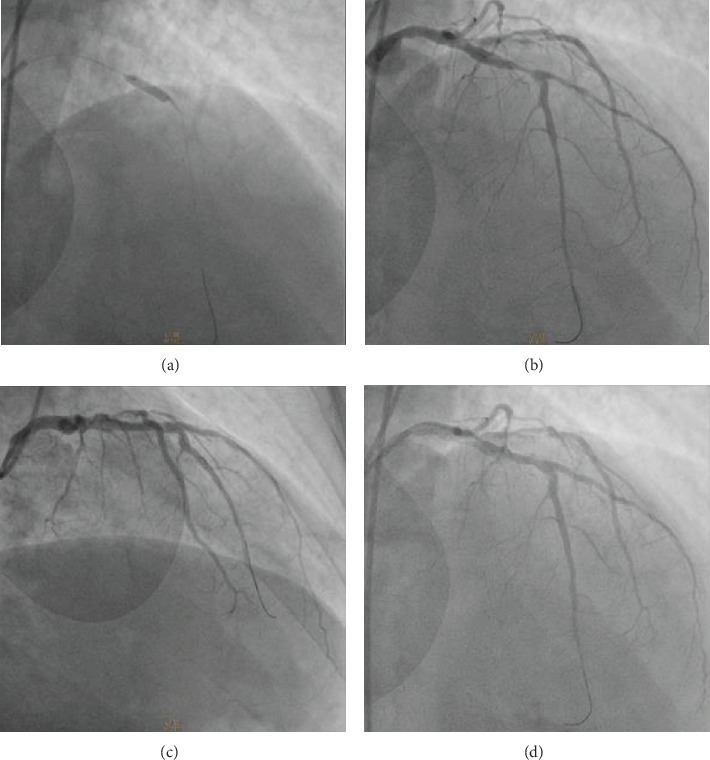
Left coronary angiogram in a RAO cranial projection showing (a) dilatation of the LAD culprit lesion with drug-eluting balloon, (b) post predilatation of the lesion using a SC Artimes balloon, and (c, d) successful drug-eluting balloon angioplasty with a TIMI grade II flow. TIMI = thrombolysis in myocardial infarction study group; RAO = right anterior oblique.

A 53-year-old man was admitted to a peripheral hospital with the diagnosis of acute myocardial infarction without ST elevation (NSTEMI) [4]. Due to the concomitant presence of first-diagnosed thrombocytopenia (platelet count (PLT) 50.000/*μ*L, visual estimate), it was decided to be treated conservatively with single antiplatelet therapy (clopidogrel 75 mg). Five days later, he developed an acute anterolateral myocardial infarction with ST elevation (STEMI) and was transferred to our department for primary percutaneous coronary intervention (PCI) (time from STEMI diagnosis to wire crossing ≈105 min). The patient had a history of untreated hyperlipidemia and unrecognized diabetes mellitus (*Η*bA1c = 11, 4%). On admission, his blood pressure was 100/75 mmHg and heart rate 100 beats per minute. On auscultation, first and second heart sounds were normal, and a third heart sound was audible. The lung examination was unremarkable. The 12-lead electrocardiogram revealed ST segment elevation in anterolateral and precordial leads. The peripheral blood smear revealed PLT of 55.000/*μ*L (visual estimate). A transthoracic echocardiogram demonstrated anteroapical and lateral wall hypokinesis and severely reduced systolic function (ejection fraction ≈ 35%). The patient was immediately transferred to the catheterization laboratory, where aspirin 80 mg and clopidogrel 300 mg were administered orally prior to coronary angiography. The right femoral artery was accessed with a 6 French sheath. Coronary angiography revealed a total occlusion of the left anterior descending artery (LAD), high-grade proximal stenosis in the first diagonal branch (90%), diffuse atherosclerosis of the left circumflex coronary artery (LCx), and moderate-severe stenosis (70%) in the middle of a dominant right coronary artery (RCA) (Figures 1 and 2). The LAD lesion was considered culprit, and PCI was performed. During the procedure, bivalirudin was administered intravenously. An ADROIT® Guiding Catheter XB 3.5 6F (Cordis Corporation, USA) and a BMW guide wire (Abbott Laboratories, USA) were used, and successful crossing of the total LAD occlusion was achieved. Subsequently, predilatation of the lesion using a balloon SC Artimes 1.5 × 12 mm at 16 Atm was done, resulting in a TIMI grade II flow. Subsequently, multiple dilatations of the LAD lesion with a drug-eluting balloon 3.5 × 15mm (Blue Medical Paclitaxel-Eluting Balloon at 6 Atm) were performed (Figure 3). Additionally, due to the presence of thrombotic material and no-reflow phenomenon, eptifibatide (a glycoprotein IIb/IIIa inhibitor) and adenosine were administered intracoronary. Following the procedure, the patient was treated with dual antiplatelet therapy (DAPT), aspirin (100 mg/day), and clopidogrel (75 mg/day), but four days later, aspirin was discontinued due to a platelet fall (from 52.000/*μ*L to 16.000/*μ*L). No minor or major bleeding was detected. Meanwhile, by requested hematology consultation and through examination of peripheral blood smear, exclusion of alternative disorders and bone marrow findings, the diagnosis of ITP was made (Table 1) [5–7]. Recommended ITP treatment included the intravenous infusion of *γ*-globulin (IG) for three days and the administration of steroids (methylprednisolone, initially 60 mg/day and subsequently 40 mg/day) as well as romiplostim (500 mcg sc weekly), to increase platelet count (Table 2).

Seven days following the procedure, the patient was transferred to the Hematology clinic, where a continuous rise of platelet count until the sixteenth day at the level of 115.000/*μ*L was observed, and the patient was discharged from the hospital stable and asymptomatic. The follow-up visit for the cardiac reevaluation of the patient was scheduled 30 days postdischarge. Treatment at discharge included methylprednisolone (40 mg/day), clopidogrel (75 mg/day), bisoprolol (5 mg/day), atorvastatin (40 mg/day), ramipril (2.5 mg/day), eplerenone (50 mg/day), furosemide (40 mg/day), ivabradine (5 mg/day), metformin (850 mg/day), and insulin glargine (40 U/day).

20 days after his discharge, the patient was readmitted to the Hematology clinic due to lung infection. During his hospitalization, he became septic and died a few days later.

## 3. Discussion

ITP is an autoimmune disorder, most commonly seen in females. It is characterized by a low platelet count due to autoantibody-mediated platelet destruction and suppression of platelet production that predispose to bleeding. Given the critical role of platelets in atherothrombosis, the prevalence of coronary artery disease in ITP seems to be low. However, ITP may be associated with increased risk of thrombotic events, including acute coronary syndromes (ACS) that may be related to endothelial damage caused by antigenic mimicry between larger and more adhesive platelets and endothelial cells. In addition, it can be related to ITP treatment with steroids that induce metabolic changes and an hypercoagulable state, or intravenous immunoglobulin that may result in expansion of plasma volume and increase in blood viscosity [8].

In general, the optimal management of STEMI includes timely revascularization of the culprit lesion with PCI and stent (s) implantation, followed by DAPT (i.e., acetylsalicylic acid and a thienopyridine derivative). However, antiplatelet therapy is not recommended, and avoidance of PCI is advised when the platelet count is below 50.000/*μ*L, due to high bleeding risk [1].

So far, no recommendations exist regarding the selection and duration of antiplatelet therapy and whether or not to implant a stent in the culprit vessel, as relatively few cases of PCI have been reported in ACS patients and concomitant ITP [3]. In ACS patients with thrombocytopenia undergoing PCI, second-generation drug-eluting stents are preferable to bare-metal stents, and radial approach is generally preferred to femoral, due to evidence of a lower risk of bleeding, as well as easy hemostatic compression [1, 9]. Due to limited data, in stented patients with ACS who have a platelet count of ≤100.000/*μ*L but ≥50.000/*μ*L, DAPT with aspirin and clopidogrel is recommended for short time—1 month—followed by a single antiplatelet agent, with clopidogrel being the treatment of choice [10]. Less is known regarding stentless PCI, although a strategy of single antiplatelet treatment has been proposed [2].

Furthermore, periprocedural supportive treatment to increase platelet counts, platelet transfusion, intravenous IG, and steroids has been used to reduce bleeding complications [8].

In our case, we decided to proceed with primary PCI using a drug-eluting balloon, as we could not predict the patient's tolerance and response to prolonged DAPT required following stent implantation, given that the patient was recently diagnosed with thrombocytopenia. The stentless approach is also supported by the recent REVascularization with paclitaxEL-coated balloon Angioplasty versus drug-eluting stenting in acute myocardial infarcTION (REVELATION) trial which demonstrated that in the setting of STEMI, the drug-coated balloon strategy was noninferior to drug-eluting stent in terms of fractional flow reserve assessed at 9 months and seemed to be safe and feasible [11]. Regarding the access site of the procedure, despite the theoretical advantages of the radial, excellent results have also been reported with the transfemoral approach [3]. Notably, in patients undergoing primary PCI without cardiogenic shock who are treated with bivalirudin (as in our case), the differences in mortality or bleeding rates related to the access site are negligible [12]. Bivalirubin, a direct thrombin inhibitor, was used as an alternative to heparin since it is the only agent proved to be safe in cases with thrombocytopenia [13].

The no-reflow phenomenon that the patient suffered is a devastating complication, and the results of several pharmacological therapies are generally unsatisfactory. In our case, adenosine and the glycoprotein IIb/IIIa inhibitor eptifibatide were administered, as the intracoronary use of the latter appears to be safe [14, 15].

In ITP patients with STEMI, primary PCI with drug-eluting balloon angioplasty is a reasonable approach that can be safely and successfully applied, if platelet count and bleeding risk are carefully monitored.

## Figures and Tables

**Table 1:** Differential diagnosis of idiopathic thrombocytopenic purpura.

Idiopathic (unknown etiology)
Pseudothrombocytopenia
Renal or liver disease
Myelodysplastic syndrome, acute leukemia
Aplastic anemia
Genetic diseases that cause thrombocytopenia
Thrombotic thrombocytopenic purpura
Heparin-induced thrombocytopenia
Infection (i.e., HIV, HCV, and Helicobacter pylori)

HIV: human immunodeficiency virus; HCV: hepatitis C virus.

**Table 2:** Platelet count and idiopathic thrombocytopenic purpura treatment during hospitalization.

	1^st^ day post PCI	2^nd^ day post PCI	3^rd^ day post PCI	4^th^ day post PCI	5^th^ day post PCI	6^th^ day post PCI	16^th^ day post PCI^∗^
PLT	52.000	32.000	47.000	56.000	16.000	22.000	115.000
Antiplatelet therapy	Aspirin Clopidogrel	Aspirin Clopidogrel	Aspirin Clopidogrel	Aspirin Clopidogrel	Clopidogrel	Clopidogrel	Clopidogrel
ITP treatment	IVIG	IVIG	IVIG			Steroids Romiplostim	Steroids

^∗^Hospital discharge. PCI: percutaneous coronary intervention; PLT: platelets; ITP: idiopathic thrombocytopenic purpura; IVIG: intravenous immunoglobulin.

## Abbreviations

ITP: Idiopathic thrombocytopenic purpura
ACS: Acute coronary syndromes
STEMI: ST elevation myocardial infarction
NSTEMI: Non-ST elevation myocardial infarction
PCI: Percutaneous coronary intervention
LAD: Left anterior descending artery
LCx: Left circumflex artery
RCA: Right coronary artery
DAPT: Dual antiplatelet therapy
IVIG: Intravenous immunoglobulin.
